# Primary palliative care in low- and middle-income countries: A systematic review and thematic synthesis of the evidence for models and outcomes

**DOI:** 10.1177/02692163241248324

**Published:** 2024-05-01

**Authors:** Anna Peeler, Oladayo Afolabi, Michael Adcock, Catherine Evans, Kennedy Nkhoma, Dorothee van Breevoort, Lindsay Farrant, Richard Harding

**Affiliations:** 1 King’s College London, Florence Nightingale Faculty of Nursing, Midwifery & Palliative care, Cicely Saunders Institute, London, UK; 2 Kamuzu University of Health Sciences, Department of Family Medicine, Blantyre, Malawi; 3 University of Cape Town, Cape Town, South Africa

**Keywords:** Palliative care, primary health care, developing countries

## Abstract

**Background:**

Serious health-related suffering is predicted to double in low- and middle-income countries by 2060. Primary care offers the best opportunity to meet Universal Health Coverage in an equitable way. Primary palliative care growth should be evidence-based to ensure provision is feasible, acceptable and culturally congruent.

**Aim:**

To identify the current evidence related to primary palliative care and to describe how primary palliative is defined in this setting, dominant typologies of care and meaningful outcome measures in LMICs.

**Design:**

A systematic review and thematic synthesis was conducted. We described the nature, extent and distribution of published literature on primary palliative care in low- and middle-income countries, use thematic synthesis to characterize typologies of primary palliative care and design a process model for care delivery in low- and middle-income countries.

**Data sources:**

Medline, Psychinfo, Global Health, Embase and CINAHL.

**Results:**

Thirty-five publications were included. Nearly half took place in Asia (*n* = 16, 45.7%). We identified five dominant typologies of primary palliative care, including delivery in primary care clinics by multidisciplinary healthcare teams and palliative care specialists, in people’s homes by healthcare professionals and volunteers and in tertiary healthcare facilities by generalists. We designed a process model for how these models operate within larger health systems and identified barriers and facilitators to implementing primary palliative care in this context.

**Conclusion:**

Evidence supporting primary palliative care in low- and middle-income countries is limited, and much of the published literature comes from Asia and southern Africa. Health systems in low- and middle-income countries have unique strengths and needs that affect primary palliative care services that should guide how services evolve to meet future need.

**What is already known about this topic?**

- Serious health-related suffering is predicted to more than double in low- and middle-income countries (LMICs) by 2060.
- Primary care is ‘widely regarded as the most inclusive, equitable and cost-effective way to achieve universal health coverage’ and should include palliative care and symptom management according to the World Health Organization.
- Much of the primary palliative care literature centres around health systems in high-income countries and falls short of considering the unique strengths and challenges of health systems in LMICs.

**What does this paper add?**

- This systematic review and thematic synthesis provides us with a framework of models of primary palliative care, a process model for how these models operate within the larger health systems of LMICs, appropriate outcomes to measure to determine service quality and efficacy and barriers and facilitators to implementing primary palliative care in this context.
- Primary palliative care is delivered in various ways in LMICs, including in primary care clinics by multidisciplinary healthcare teams and occasionally palliative care specialists, in people’s homes by healthcare professionals and volunteers and in tertiary healthcare facilities by generalists. These models are shaped by historical events, government policies, resource availability, cultural norms and community networks.
- Primary palliative care literature focussed on LMICs is limited, particularly in the central and South Americas and parts of Africa. Published literature likely does reflect all primary palliative care services that are delivered in LMICs.

**Implications for practice, theory or policy**

- The lack of a clear definition of what primary palliative care means in LMICs can limit uptake and availability.
- Future research should focus on how to sustainably staff and fund primary palliative care services, how to effectively train and mentor staff to deliver these services to meet the growing need, how to best conduct holistic assessment and achieve goal-concordant care LMICs and how to enable patients with life limiting illness to remain at home.

## Introduction

As the global population ages, death with serious health-related suffering is predicted to more than double in low- and middle-income countries (LMICs) by 2060.^1,2^ This represents over 20 million people experiencing preventable suffering at the end of life. Palliative care improves outcomes for patients living with life limiting illness and their families, ameliorating health-related suffering.^3^ Early integrated palliative care has been shown to improve costs, physical and psychological symptom burden, quality of life, home death rates and survival time for many life-limiting illnesses.^4–7^ Even so, only 10% of the 20 million people each year who require palliative care actually receive it. Though most provision is in high income countries, 80% of need is in LMICs.^8^ Following the Universal Health Coverage mandate to include palliative care in essential health services and the Lancet Commission on Pain & Palliative Care, the World Health Organization has tasked health systems to incorporate palliative care and symptom relief into primary care to improve access.^9,10^

Primary care broadly refers to healthcare delivered in the community as a first line access point for individuals to engage with the healthcare system.^11^ It is meant to provide people with integrated health services, from health promotion to disease prevention, treatment, rehabilitation and palliative care across the lifespan. According to the World Health Organization, primary care services offer the best opportunity to meet Universal Health Coverage goals in a sustainable and equitable way.^12,13^

Primary palliative care services provided by primary practitioners or in the primary care setting has the potential to help fill the gap between need and delivery.^14^ This care is provided by general practitioners in any setting or is provided by specialists operating in a generalist capacity.^15^ Primary palliative care research in high-income countries has been increasing over the last few decades, but research in LMICs is expanding more slowly.^16^ Primary care and palliative care delivery varies hugely between countries, health systems and communities due to differences in resource availability and allocation, local policies and cultural norms.^9,17–19^ Therefore, there is a need to understand how primary palliative care is delivered across diverse settings, what works well, what models of care might be worth adapting and replicating, what gaps exist and what learning can be shared between the global north and south. To fill this gap, the aim of this review is to identify the current evidence related to primary palliative care and to describe how primary palliative is defined in this setting, dominant typologies of care and meaningful outcome measures in LMICs. Our research questions were: (1) What published literature related to primary palliative care in LMICs exists? (2) How is primary palliative care defined in this setting and what are the dominant typologies of care? (3) How is palliative care delivered within primary care? (4) How are these models of care being evaluated and what outcomes are meaningful? (5) What are the barriers and facilitators to incorporating palliative care into primary care in LMICs?

## Methods

### Study design

This systematic review and thematic synthesis was conducted in order to identify and systematically synthesize the published evidence on primary palliative care in LMICs.^20^ Using thematic synthesis methodology as a means of analysis in systematic reviews is well established in the palliative care literature.^21–23^ All data are reported according to the Preferred Reporting Items for Systematic Reviews and Meta-Analyses (PRISMA) guidelines.^24^

### Search strategy

**Figure 1.**
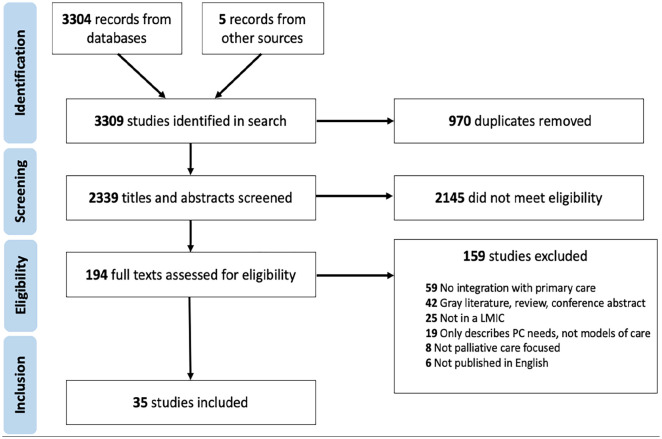
PRIMSA flowchart.^32^

With input from an interdisciplinary team of researchers, clinicians and key stakeholders, a search strategy was developed based on previous palliative and primary health care literature.^25–27^ We searched five bibliographic databases, Medline, Psychinfo, Global Health, Embase and CINAHL from inception to January 2023 (see inclusion and exclusion criteria in Figure 1) and removed any duplicates. Low- and middle-income countries were defined based on the 2020 World Bank definition.^28^ The search terms are reported in Appendix 1. Additionally, we hand searched key journals and reference lists from related reviews for additional studies.

### Study identification and selection

Three reviewers (AP, OA and MA) independently reviewed each of the articles identified in the search. Studies were appraised against the inclusion and exclusion criteria first based on their title and abstract, then those retained were subsequently appraised based on their full text. Studies were included if they (1) focussed on palliative or hospice care or care delivered in the last year of life for people with life-limiting conditions and/or carers, (2) took place in the primary care setting OR focussed on care delivered by general practitioners or family physicians, (3) took place in a low-, lower middle- or upper middle-income country or countries as defined by the 2019 World Bank classification levels,^28^ (4) were peer-reviewed and reported empirical, primary research (any methodology) and (5) were written in English. Studies were excluded if (1) they solely focussed on bereavement care (after the patient has died), (2) took place in-hospital, other acute care setting or long-term care facility, (3) focussed on care delivered only by family members or untrained carers, or (4) less than 50% of participants came from a LMIC. Any conflicts were discussed with the study team and decided by consensus.

### Data extraction

Salient variables in line with the research questions and the guiding methodology were extracted to a purpose-built excel spreadsheet. These included the authors, year of publication, setting, disease focus, aims and objectives, study design, intervention components (if applicable), description of the model of care, inclusion and exclusion criteria, study participants, outcome measures and data collected, relevant findings, mechanism of action (if reported), facilitators and barriers to palliative care incorporation into primary care and study limitations. Each was extracted from the full text of the included articles by a member of the study team (AP, OA and MA). Facilitators, barriers and study limitations were identified based on factors defined by the papers’ authors.

### Quality appraisal

The quality of included studies was assessed using the Mixed Methods Appraisal Tool.^29^ This tool uses screening questions and methodology specific quality criteria to systematically appraise bias and rigour in five different methodologies, (1) qualitative, (2) quantitative randomized controlled trials, (3) quantitative non-randomized, (4) quantitative descriptive and (5) mixed methods. Quantitative only and qualitative only studies are scored on a scale from 0 to 5, with 5 meaning that 100% of the quality criteria are met. Mixed methods studies are assessed based on their quantitative component, the qualitative component, then on the mixed methods integration, each scored on a scale from 0 to 5. The overall score for mixed methods studies is the lowest score of any of the components, as overall quality cannot be more than the weakest component. Quality was assessed by one member of the review team (AP) and checked by another (OA).

### Summarizing, synthesizing and reporting results

We began by describing the extent, nature and distribution of the charted data. To do this, we geographically mapped the retained studies, summarized and compared the models of primary palliative care described, collated outcome measures used to evaluate services and outlined the barriers and facilitators to primary palliative care integration reported in each study.

Next, a modified thematic synthesis of the models of care in each included study based on Thomas and Harden’s methods was undertaken to understand how palliative care is being delivered within primary care.^20^ Descriptive themes were developed by inductively coding the care model summaries to identify common elements such as where the care takes place, who delivers care and funding mechanisms. From these descriptive themes, we applied a conceptual typological framework that sought to characterize modes of palliative care delivery within primary care.^30^ Identifying typologies allowed us to distill common components, detect patterns and compare across settings to gain conceptual clarity about primary palliative care delivery methods employed in LMICs.

While typologies are useful in characterizing general care patterns and methods of integrating palliative care into primary care, they can fall short of recognizing the unique ecosystem in which each operates or how they interact with the larger healthcare system.^30^ For this reason, we developed a system-based model that incorporates the five identified typologies of integration while also acknowledging the larger healthcare system described in the included literature and how bespoke services meet patients’ and families’ needs.^31^

Codes and themes were independently identified by the three reviewers (OA, AP and MA). Disagreements adjudicated by the wider research team. To ensure credibility, resulting codes and themes were also discussed and checked for reliability through ongoing testing and review by members of the wider research team.

## Results

### Search yield

As seen in Figure 1, the electronic search yielded 2334 results after de-duplication. Five additional studies were identified from other sources (i.e. Reference lists from related reviews, expert referral). Of those, 2145 were excluded at title and abstract screening, and 159 excluded at full paper review. In total, *n* = 35 articles were included in the final analysis.

### Scope of included studies

**Figure 2.**
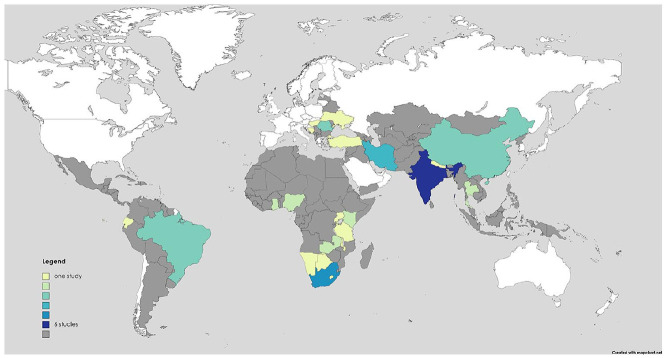
Map of study settings.

Study summaries are presented in Supplemental Table 1. Of the 35 included studies, *n* = 16 were conducted in Asia (45.7%), *n* = 9 in Africa (25.7%), *n* = 6 in Europe (17.1%) and *n* = 4 in the Americas (11.4%). A map of included studies can be seen in Figure 2. With respect to evaluation design, *n* = 9 (25.7%) studies were experimental (*n* = 8 not randomized, *n* = 1 randomized controlled trial); *n* = 26 (74.2%) studies were observational (*n* = 16 prospective, *n* = 10 retrospective); The study methods were: *n* = 15 (42.8%) qualitative data collection only, *n* = 4 (11.4%) used mixed methods. In terms of population studied, *n* = 14 (40%) included only patients with cancer, *n* = 4 (11.4%) with HIV/AIDS, *n* = 3 (8.5%) elderly or home-bound patients and *n* = 1 (2.9%) with multimorbidity.

### Quality appraisal

Of the 35 included studies, 15 (42.8%) were qualitative only, 1 (2.9%) was a quantitative randomized controlled trial, none were quantitative non-randomized, 15 (42.8%) were quantitative descriptive and 4 (11.4%) were mixed methods. In total, 13 (37.1%) studies met five of their methodology specific quality indicators, 9 (25.7%) met 4, 11 (31.4%) met 3 and 2 (5.7%) met 2. No studies met one or zero quality criteria.

Most (10 of 13) of the highest quality studies^33–45^ were qualitative only. Both of the lowest quality studies^46,47^ were quantitative descriptive. For the mixed methods studies, *n* = 3 studies rated poorly on the consideration of the divergences and inconsistencies between quantitative and qualitative results within their integration.^48–50^ The majority of the qualitative studies and the qualitative sections of the mixed methods studies were rated highly. However, coherence between qualitative data sources, collection, analysis and interpretation was identified as an issue in *n* = 6 studies. Of the quantitative studies and quantitative sections of the mixed methods studies, *n* = 9 studies were graded for low risk of non-response bias as this information was not consistently reported across the papers. There were no patterns observed in study quality based on geographic location, disease focus or typologies of primary palliative care presented. A detailed look at the quality criteria that each included study met can be found in Supplemental Table 2. We included all studies in our analysis.

### Typologies of primary palliative care

From the models of care described in the included studies (seen in Supplemental Table 1), we identified five typologies that characterized modes of primary palliative care delivery. These typologies differ based on where the care takes place, who is involved in delivering the care and their training requirements, where the clinical team is based, the types of services that are offered and from where and how they are funded.

**Figure 3.**
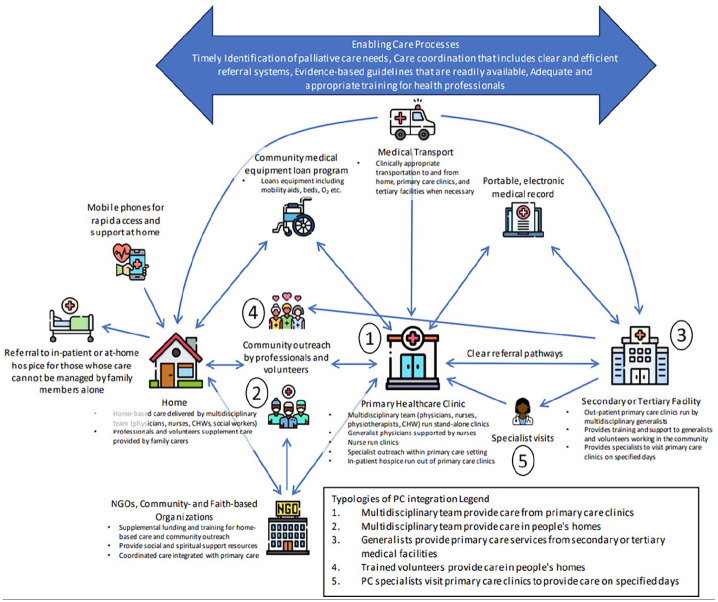
Systems-based approach to palliative care integration into primary care in low- and middle-income countries.

Nineteen (54.3%) studies described a model in which multidisciplinary teams of generalists (made up of various combinations of nurses, physicians, community health workers, social workers, physiotherapists and pharmacists) delivered palliative care services in primary care clinics (labelled 1 in Figure 3). Healthcare workers received differing levels of specific training in palliative or end-of-life care, though most often, they received little to none. Of the studies that explored their confidence in delivering palliative services, all found that generalists felt unprepared to deliver adequate palliative and end-of-life care such as communicating a terminal diagnosis,^51–54^ navigating goals of care conversations,^39,53^ titrating opioids,^41,46,51–56^ managing complications,^40,45,51,55^ providing psychosocial and spiritual support to families,^39,41,45,57,58^ managing the emotional burden of caring for dying patients^37,58^ and coordinating end-of-life care.^45,55^ Services provided include discussion of diagnosis and development of care plans, symptom assessment and management with a particular focus on pain relief, nutrition support, advance care planning, medication optimization, chronic disease management, carer education and bereavement services. These clinics are often financed by governments, private insurance or out-of-pocket expenditures. In the included studies, this typology was seen in Bosnia-Herzegovina,^54^ Botswana,^59^ Brazil,^40,57^ China,^53^ Ecuador,^41^ Ghana,^56,60^ India,^38,46^ Iran,^48,61,62^ Kenya,^56^ Lesotho,^59^ Namibia,^59^ Nepal,^52^ Nigeria,^34,56^ South Africa,^44,56,59,63^ Tanzania,^56^ Thailand^51^ and Zambia.^33,56^

Twenty-one (60%) described a method of integration in which multidisciplinary teams of generalists deliver home based care (labelled 2 in Figure 3), financed by public and private means. This could be provided in conjunction with clinic- or healthcare facility-based care or as a stand-alone service. Again, some healthcare workers providing home-based care received some palliative care training, but many received none. Services described in the studies included symptom assessment and management, homeopathic or traditional healing practices with a focus on spiritual support, rehabilitation services, medical equipment allocation, coordination with and referral to other healthcare providers and community volunteers, physical and emotional support throughout the dying process for both patients and family members, medication delivery and management, nutrition support, basic nursing tasks (i.e. bathing, dressing changes, continence management), carer education and bereavement support. This typology was seen in studies in Bosnia-Herzegovina,^54^ Botswana,^59^ Brazil,^40,64^ China,^39,45^ India,^38,42,43,65,66^ Lesotho,^59^ Malawi,^37^ Namibia,^59^ Nepal,^52^ Romania,^36,55,58^ South Africa,^44,59,63^ Turkey,^47^ Ukraine^67^ and Zambia.^33^

Ten (28.6%) studies described a model of care in which generalists, including family physicians, general practitioners and hospitalists, delivered palliative care services based in hospitals and other secondary or tertiary healthcare settings (labelled 3 in Figure 3). In this typology, generalists may have some palliative or end-of-life training, but often they have very little. Generalists provide first point of contact care that includes diagnosis of life-limiting illness and development of care plans, referral to surgical intervention or specialist care if necessary, symptom assessment and management with a focus on pain relief, advance care planning, medication optimization, chronic disease management and carer education. This typology was seen in studies conducted in Bosnia-Herzegovina,^54^ China,^39,45^ India,^46^ Iran,^49^ Malawi,^37^ Nigeria,^34^ South Africa^44^ and Thailand.^36,51^

Seven studies (20%) described a model of care in which volunteers from within the community provide home-based care including referral to healthcare facilities for those with complex needs, basic nursing tasks, nutrition support and social, emotional and spiritual support for patients and families throughout the illness trajectory (labelled as 4 in Figure 3). This often arose out of necessity in communities with very little access to formal palliative care services. In this typology, volunteers are typically supervised by healthcare professionals operating out of primary care clinics or tertiary medical facilities with referral pathways for specialist care for patients with complex needs or situations. This typology was seen in studies conducted in two Indian states^38,43,65,66^ and Sub-Saharan African countries of Botswana,^59^ Lesotho,^59^ Namibia^59^ and South Africa.^35,59,63^

Lastly, two studies (5.7%) described a pathway in which palliative care specialists occasionally provided services in the primary healthcare setting, often as a consult service available at the clinic on specified days (labelled 5 in Figure 3). Though specialist palliative care providers were delivering the care, authors described this as primary palliative care because they were operating out of a primary care or community setting and were the patient’s entry point into the healthcare system. Patients with palliative needs are scheduled to come to clinics when the specialist is working so their needs can be addressed. Specialists provide physical and emotional support for patients and families with complex needs, advanced medication optimization and management of life-threatening complications such as neutropenia, infection, inability to eat, pressure wounds and delirium. This typology is often funded by governments, private insurance, out-of-pocket or by non-government organizations (NGOs). This typology was seen in studies conducted in India^46^ and Turkey.^47^

The novel process model seen in Figure 3 represents an operationalization of primary palliative care delivery taking into account the five typologies identified in this review as well as contextual information from the included studies affecting palliative care uptake, referral pathways, specific services unable to be characterized and physical and human resource supply points.

#### Outcome measures

The quantitative outcome measures used to evaluate primary palliative care services in the included studies can be seen in Table 1. *N* = 7 studies included at least one palliative care-specific measure like the Palliative care Outcome Scale, symptom prevalence or severity (i.e. pain and depression) or quality of life outcomes like QoL Questionnaire, Health Related Quality of Life and the EuroQoL. *N* = 6 examined patient and caregiver experience outcomes using measures like the Patient Experience Questionnaire or the Zarit Caregiver Burden scale. Lastly, *n* = 12 included implementation focussed outcomes such as recruitment and retention rates, intervention fidelity and measures of service utilization (i.e. type and frequency of home-care visits, services provided and medication dosages). No studies measured social outcomes such as social support, discrimination or stigma or financial variables like financial strain, out-of-pocket expenditure or economic evaluation of services.

**Table 1.** Outcome measures.

Outcomes	Outcome measures
Palliative care outcomes	African Palliative Outcome Scale^34,60^
	Prevalence of symptoms^36^
	Intensity of symptoms^36^
	Primary Care Screening Questionnaire for Depression^65^
Attitudes and/or knowledge of Palliative Care	Bradley Attitude Questionnaire^53^
	Death Attitude Profile^53^
	Novel survey to assess healthcare workers attitudes and knowledge^41,46,47,49,51,53–55,58,62^
	Novel survey to assess patient/carer attitudes and knowledge^33,56^
Performance and function focussed outcomes	Karnofsky Performance Scale^57^
	Activities of Daily Living^34,50^
	Functional status^36^
Quality of life outcomes	QoL Questionnaire^67^
	Health Related QOL^43,59^
	EuroQoL^36,65^
	Medical Outcome Scale – HIV^60^
Patient experience outcomes	CARE Measure^60^
	Patient Experience Questionnaire^60^
	Satisfaction^67^
	Positive Outcomes Scale^60^
Carer focussed outcome	Family empowerment^42,68^
	Zarit Caregiver Burden^69^
	Family Needs Questionnaire^43^
Intervention development and implementation outcomes	Recruitment and retention rates^60^
	Intervention fidelity^52,60^
	Intervention acceptability^60^
	Adherence to treatment plan^50,59^
	Service utilization^36,38,41,43,48,50,52,54,57,63^
	Effect size^60^

### Barriers and facilitators to primary palliative care delivery

Table 2 shows the barriers and facilitators to the delivery of primary palliative care as identified by the authors of the included studies. Based on our narrative analysis, each can be categorized as: (1) funding and physical resources, (2) human resources and training, (3) cultural norms, (4) statutory guidelines, (5) processes and (6) networks and communities. The most common barriers described were funding and resources limitations (*n* = 13, 37.1%), misconceptions about palliative care related to language and translation (*n* = 11, 31.4%), insufficient palliative care education for primary healthcare providers leading to gaps in knowledge (*n* = 7, 20%) and government policies that prioritize curative treatment over holistic care (*n* = 4, 11.4%). The most common facilitators were robust primary healthcare workforce supplemented by well-trained CHWs (*n* = 11, 31.4%), strong connections with local and international NGOs (*n* = 5, 14.3%) and established community networks that support palliative care delivery, volunteers and information provision (*n* = 5, 14.3%).

**Table 2.** Barriers and facilitators to primary palliative care integration.

Category	Barriers	Facilitators
Funding and physical resources	• Lack of palliative care resources and supplies including opioid medications^35,37,46–48,52,62,63,65^ • Waning donor funding for HIV/AIDS work^33^ • Shortage of appropriate facilities^62^ • Distance from services^35,37,52,62,66^	• Electronic health record system that supports coordinated care^43,61,62^
Human resources and training	• Lack of palliative care training for generalists creating gaps in skills and knowledge related to truth telling, pain control and management with morphine, emergency management in terminal cancer care and treatment of fluid intake in terminal stages, treatment of complications^38,46,47,51,58,61,62^ • Interprofessional distrust^34^ • Healthcare providers lacked confidence in providing palliative care services because of patient complexity, inadequate training and insufficient resources^34,46,51,55,62^ • Difficulty retaining volunteers^37^ • Tendency of GPs to claim palliative services are ‘out of our scope’^34,46^ • No dedicated workforce and transport for home-based care^38^	• Availability of GPs or family physicians^46,48,51,61^ • Community health workers providing referral to healthcare provider at the facility, providing physical and spiritual comfort, educating patient-family and liaising between them and the healthcare team and supporting medication management^35,38,44,52,66^ • Volunteer workers’ willingness to help support palliative care patients, their inclination to train in palliative care and enthusiasm to refer to guidelines while caring for patients^46,52^ • Communication skills training^34,46,52,53^
Cultural Norms	• Misconceptions about palliative care ^34,35,40,42,46,49,50,52,53,55,60,61^ • Culture of withholding information about diagnosis from patient and only telling family^39,45,51,53^ • Paternalistic healthcare culture that discourages individual agency and self-efficacy necessary for person-centred care decisions^34,51,55^ • Stigma associated with living with incurable illness^47,52^	• Community understanding of palliative care and available services^35,48,49^
Statutory guidelines	• National health policy that focusses on curative treatment rather than holistic care^34,53,57,60^ • Legal and administrative limitations (e.g. fear of being blamed for the death of the patient)^52,61^ • Lack of palliative care services integration within health tariffs and insurance coverage leading to insufficient reimbursement for palliative care services^61^	• Political buy-in^35,38,48,49^ • National palliative care policy including funding, priorities and guidelines for healthcare workers • Legal authorization and clear guidelines on opioid use^41,51,52^
Processes	• Changing burden of disease that leads to a need for new models of care^33,50^ • Lack of standards and guidelines for identification of palliative care needs, referral to and provision of primary palliative care ^43,46,50,62^ • Unclear role delineation in healthcare settings^34,44,46^ • Poor communication between healthcare teams, patients and families^46,51,52,58^ • Late referral to palliative care ^36,38,50,58^ • Lack of integration of the different health care streams^34,37,59^ • Centralized opioid management and distribution approach makes medicines access for pain control difficult^48,61^	• Established referral system for coordination of services with specialists^46,48,49,63^ • Support to maintain health workers’ personal wellbeing^52^ • Embedding traditional knowledge with modern practice (i.e. integration of biomedicine with indigenous healing traditions, using mobile technology to facilitate communication)^35,37,38^ • Empowering family caregivers through the provision of appropriate training and communication^37,62^ • Reverse referral of patients from subspecialty centres to primary care services^48^
Networks and community	• Palliative care services located far away from community centres^37,56^	• Having strong network with local NGOs^33,49,56,59,62^ • Capitalizing on community mutual support and cultural values^33,35,44,49,66^ • Existing trust and respect between communities and primary healthcare centres^34,60^

Examining the facilitators and barriers from a geographic perspective illuminated certain trends. Barriers, such as physical and human resource limitations, opioid availability and misconceptions about palliative care, were commonly discussed in reports from every continent. Similarly, facilitators such as a robust, well-trained workforce of generalists and political buy-in were discussed in studies from around the world. Even so, there were a few barriers that were concentrated in specific geographic areas, including a culture of withholding diagnosis from the patient in east Asia and a lack of referral pathways in sub-Saharan Africa. Furthermore, some facilitators were focussed in specific regions, such as strong networks of community health workers and embedding traditional medicine with modern practice in South Asia and sub-Saharan Africa.

## Discussion

To our knowledge, this review is the first to identify and describe the published evidence reporting research into primary palliative care in LMICs worldwide. Just nine studies (25.7%) evaluated the primary palliative care models in LMICs. This represents a significant gap in the literature and signals a need for more robust research evaluating efficacy and feasibility of primary palliative care models in LMICs.

We found that much of the published literature comes from Asia and southern Africa (particularly from India, China, Iran and South Africa). The Americas and parts of northern and central Africa are underrepresented in the published literature. This reflects the level of palliative care development reported in other research.^70,71^ By 2017, India, China, Iran and South Africa had achieved generalized palliative care availability throughout each country according to the World Health Organization classification. Conversely, no LMICs in central or South America had achieved advanced integration of palliative care, except Costa Rica. Similarly, Malawi and Swaziland were the only LMICs in Africa to have achieved advanced integration, while nearly every African nation with no published primary palliative care literature had no known palliative care activity or was in very early stages of capacity building.^71^ As such, there is likely some correlation between palliative care development and published literature on the subject of primary palliative care reported from that country.

### Typologies

From the included studies, we identified five typologies of care that characterize how palliative care is being delivered within primary care settings or by primary care practitioners in LMICs. The most prevalent typology was (1) multidisciplinary teams made up of nurses, physicians, community health workers, social workers, physiotherapists and pharmacists delivering palliative care services in primary care clinics, followed by (2) multidisciplinary teams providing care in patients’ homes, (3) generalists providing primary palliative in in tertiary medical facilities, (4) volunteers providing care at home and occasionally and (5) palliative care specialists providing services in the primary healthcare setting. These typologies echo the World Health Organization’s six models of integrating public health and primary care functions, though they reflect important nuances specific to the context of integrating palliative care in LMICs.^72^

Examining the included studies in the context of the larger literature base, primary palliative care is poorly defined and can look very different in LMICs as compared to high-income countries despite similar ethos and priorities. While some of the typologies we identified can been seen in high-income countries, others are less prevalent or are typically discussed in high-income settings using different terminology. For example, typologies one and two, multidisciplinary teams providing palliative care services in primary care clinics and patients’ homes, respectively, are well documented in the literature from high-income countries.^16^ Typologies three, generalists providing primary palliative in in tertiary medical facilities and four, volunteers providing care at home, are less described in the literature from high-income countries and reflect the need in LMICs to deliver care pragmatically where patients engage with the healthcare system in the context of severe resource limitations.^73^ Finally, typology five, palliative care specialists occasionally providing services in the primary healthcare setting, was identified as primary palliative care in two studies included in this review but is often characterized as ‘specialist outreach’ in high-income settings.^74^

Overall, the differences in the delivery of primary palliative care are shaped by factors including who delivers the care and how, how primary palliative care developed, funding, the predominant disease burden, language and cultural beliefs. In this setting, generalists with no palliative care training may be the only available resource for delivering palliative care services, and often, they deliver primary palliative care from whatever setting they are already operating.^75^ Furthermore, resource limitations may limit the specialist palliative care providers operating at the primary healthcare level. Where high-income countries typically view primary healthcare as stand-alone clinics with care provided by medical professionals,^15^ in LMICs entry point care can be provided out of hospitals or in the home by community health workers (with varying degrees of training), lay volunteers or traditional healers.^69^ Moreover, the preponderant disease burden can be different in LMICs compared to high-income countries, with HIV/AIDS, cancers related to infectious diseases (e.g. cervical cancer) and tuberculosis having a bigger impact than in high-income countries.^76^ Even among LMICs, needs vary by region, demographics and context.^77^ These contextual differences and divergence in cultural norms between high-income countries and LMICs require a different approach to the provision of primary palliative care services and might include different metrics for success. As such, models of primary palliative care developed in high-income countries may not be effective in LMICs. Even so, health systems in high-income countries and LMICs can learn from each other’s successes and challenges.

The novel process model for primary palliative care in the included studies is shaped by the needs and context of individual communities. Many of the included studies describe services that evolved from initiatives started by volunteers, community organizations or religious institutions that emerged out of necessity during crises such as the AIDS epidemic, natural disasters or ongoing disease outbreaks.^33^ These services continued after the crisis ended and transitioned to part of standard care because people and governments appreciated their continuing utility. For this reason, primary palliative care has developed differently in LMICs as compared with high-income countries, and as such, should continue to expand and evolve based on the unique circumstances of communities there. While the process model we identified reflects only published literature identified in this review, we recognize that there are likely many more methods of incorporating palliative care into primary care currently being delivered in LMICs.

### Barriers and facilitators to primary palliative care delivery

In this review, we identified key barriers and facilitators to delivering primary palliative care in LMICs. The insufficient palliative care training for generalists discussed in many of the studies highlights a critical gap that can lead to inadequate service delivery, frustration and burnout in the healthcare workforce and worse patient outcomes. In every study where palliative care knowledge and confidence was assessed, practitioners felt unprepared to deliver appropriate care often citing the lack of training resources.^37,39–41,45,46,51–58^ While this dearth of palliative care training for generalists is not unique to LMICs, they do experience outsized resource limitations that can exacerbate training deficits and force generalists to provide extempore care.^78^ This, coupled with the stark increase in serious health related suffering expected over the next few decades, could mean that millions of people who access palliative care through their primary care provider will receive inadequate care.^1^ Conversely, studies that aimed to address the lack of palliative care training for those working in primary care proved that educational interventions and mentorship schemes that improved palliative care knowledge and confidence for generalists facilitated improve palliative care delivery.^47,56,58,60,66,67^

Furthermore, differing funding mechanisms was a key insight identified in this review. In LMICs, a lot of funding and resources for palliative and end-of-life care is provided by NGOs, charities and religious organizations, particularly when governments are unable to adequately meet the palliative care need.^79^ Strong connections with NGOs was a major facilitator to delivering high-quality palliative care. In contrast, high-income countries typically have more stable funding and resources allocated to healthcare in government budgets, mitigating the need for charities to be sole providers of care.^80^ As Sustainable Development Goal 3.8 calls for Universal Health Coverage which mandates global palliative care provision, securing sustainable funding for primary palliative care should be a priority.

Beyond logistical considerations like staffing and funding, the delivery of primary palliative care in LMICs has been shaped by government policies and cultural norms emphasizing the importance of curative healthcare. Governments and policy makers in LMICs have a tendency to prioritize a bio-medical model that focusses on diagnosis and treatments, and related metrics, rather than quality of life considerations.^81^ At times, this can contrast with the philosophy of palliative care and limit its development. Many of the models of palliative care described in this review aim to disrupt this phenomenon by employing holistic, community-based approaches to palliative and end of life care that are more attuned to the needs of community members and can adapt more readily to changing needs as disease burden shifts towards chronic disease management. This aligns with the philosophy of primary healthcare defined by multinational institutions like the World Health Organization which emphasize the need for locally informed approaches that transcend the biomedical model.^10^

Additionally, we found language to be a common discussion point in the provision of primary palliative care in LMICs. Multiple studies citied language as a barrier to successful integration of palliative care into existing primary care infrastructure.^34,50,52,53,60^ They found that some local languages do not have a direct translation for ‘palliative care’ and that this can be a significant impediment to effectively teaching communities about the benefits of palliative care and delivering services. Furthermore, some authors mentioned that they hesitated to use the word palliative care because it had the potential to spark fear in patients and family members. Moreover, even some healthcare professionals had misconceptions about what palliative care means and how it impacts the care of patients with life limiting illness.^39,40,42,55^ Consequently, these mistranslations and misconceptions about palliative care can cause healthcare workers to withhold potentially beneficial services and patients and families to refuse care.

Lastly, cultural beliefs and norms were often cited as both barriers and facilitators to integrating palliative care into primary care in LMICs. Societal stigma, health literacy, religiosity and spirituality impact preference towards palliative care.^82^ On one hand, in settings where there was strong community awareness of palliative care, there is demand for services and an environment that encourages political buy-in.^35,48,49^ Conversely, stigma and misconceptions about palliative care can impede uptake of primary palliative care.^34,52,60^ Beyond perceptions of palliative care, some authors described patients’ predisposition to accepting the advice and care plans from healthcare providers without patient or family input, which is often attributed to a ‘lack of agency’ or ‘hesitancy to actively engage’ in developing the plan of care.^34,51,55^ Authors also noted a culture of withholding information from cancer patients because talking about death and dying is considered to be disrespectful, bad luck or causes the patients and family to lose hope, primarily seen in countries in Asia.^45,51,53^ Some studies included in this review questioned whether the western model of palliative care promoted by the World Health Organization is universally compatible with other cultures.

## Limitations

The results of this review should be considered alongside a few limitations. First, due to time and resources constraints, we were unable to examine unpublished or non-peer reviewed literature or studies published in languages other than English. As such, there are likely models of primary palliative care being delivered in LMICs that have not been included in the literature that we examined. The typologies that we identified and the process model we developed only reflect the published literature as defined by the authors of the included studies. Second, both primary care and palliative care are concepts that involve a degree of subjectivity. Though we attempted to capture as many published articles on the subject as possible using definitions and search terms from previously published reviews, it is possible that some literature based on different terminology or different conceptualizations of primary care or palliative care was missed. Moreover, the included studies represented a spectrum of quality and rigour. Only 37.1% met all five of the quality indicators defined by the Mixed Methods Appraisal Tool.^29^ Lastly, the World Bank’s list of LMICs fluctuates each year. We used the 2020 definitions for the purpose of this review, and as such, the results reflect studies that were conducted in LMICs as defined in 2020.^28^

## Conclusion

Palliative and end-of-life care is a human right and achieving equitable access should be a priority. Health systems and communities in LMICs have unique needs and contextual considerations that affect the delivery of palliative care services and their integration into primary care. Cultural norms, government priorities, resource limitations, language and the rapidly increasing burden of chronic disease shape how primary palliative care has developed and should guide how it evolves to meet future need. Though it is clear that expanding access to high-quality palliative care strengthens health systems, determining the best, most efficient ways to do this in low-resource settings can be difficult.^83^ This review provides us with a preliminary list of typologies of primary palliative care in LMICs, a process model for how these typologies operate within larger health systems, appropriate outcomes to measure to determine service quality and efficacy, and barriers and facilitators to implementing primary palliative care in this context. Future research should focus on how to sustainably staff and fund primary palliative care services, how to effectively train and mentor staff to deliver these services to meet the growing need, how to best conduct holistic assessment and achieve goal-concordant care LMICs, and how to enable patients with life limiting illness to remain at home.

## Supplemental Material

sj-docx-1-pmj-10.1177_02692163241248324 – Supplemental material for Primary palliative care in low- and middle-income countries: A systematic review and thematic synthesis of the evidence for models and outcomes Supplemental material, sj-docx-1-pmj-10.1177_02692163241248324 for Primary palliative care in low- and middle-income countries: A systematic review and thematic synthesis of the evidence for models and outcomes by Anna Peeler, Oladayo Afolabi, Michael Adcock, Catherine Evans, Kennedy Nkhoma, Dorothee van Breevoort, Lindsay Farrant and Richard Harding in Palliative Medicine

## Appendix 1: Search terms

### Palliative care

PALLIATIVE CARE OR palliative therapy OR end of life OR terminal care OR supportive care OR palliat* OR hospice OR symptom* treat* OR palliat* treat* OR EOL care OR dying.

### AND primary care

Primary care nursing OR primary care OR primary health care OR primary medical care OR primary healthcare OR family practice OR general practi* OR GP OR family physician* OR family doctor* OR family practi* OR physicians, family OR physicians, primary care OR district nurs* OR community nurs* OR community health services OR communit* health.

### AND low- and middle-income country

Low-income country OR middle-income country OR low- and middle-income countries OR developing country OR LMIC OR low-resource OR Africa OR Africa South of the Sahara OR Central America OR Afghanistan OR Albania OR Algeria OR American Samoa OR Angola OR Antigua and Barbuda OR Argentina OR Armenia OR Azerbaijan OR Bangladesh OR Republic of Belarus OR Belize OR Benin OR Bhutan OR Bolivia OR Bosnia-Herzegovina OR Botswana OR Brazil OR Bulgaria OR Burkina Faso OR Burundi OR Cape Verde OR Cameroon OR Cambodia OR Central African Republic OR Chad OR China OR Colombia OR Comoros OR Congo OR Democratic Republic of the Congo OR Costa Rica OR Cote d’Ivoire OR Cuba OR Djibouti OR Dominica OR Dominican Republic OR Ecuador OR Egypt OR El Salvador OR Eritrea OR Ethiopia OR Fiji OR Gabon OR Gambia OR Georgia (Republic) OR Ghana OR Grenada OR Guatemala OR Guinea OR Equatorial Guinea OR Guinea-Bissau OR Guyana OR Haiti OR Honduras OR India OR Indonesia OR Iran OR Iraq OR Jamaica OR Jordan OR Kazakhstan OR Kenya OR Kosovo OR Micronesia OR Democratic People’s Republic of Korea OR Yugoslavia OR Kyrgyzstan OR Laos OR Latvia OR Lebanon OR Lesotho OR Liberia OR Libya OR Macedonia (Republic) OR Madagascar OR Malawi OR Malaysia OR Indian Ocean Islands OR Mali OR Mauritania OR Mauritius OR Mexico OR Moldova OR Mongolia OR Montenegro OR Morocco OR Mozambique OR Myanmar OR Namibia OR Nepal OR Nicaragua OR Niger OR Nigeria OR Pakistan OR Palau OR Panama OR Papua New Guinea OR Paraguay OR Peru OR Philippines OR Romania OR Siberia OR Rwanda OR Samoa OR Atlantic Islands OR Senegal OR Serbia OR Seychelles OR Sierra Leone OR Melanesia OR Somalia OR South Africa OR Sri Lanka OR Saint Lucia OR Saint Vincent and the Grenadines OR Sudan OR Suriname OR Swaziland OR Syria OR Tajikistan OR Tanzania OR Thailand OR East Timor OR Togo OR Tonga OR Tunisia OR Turkey OR Turkmenistan OR Micronesia OR Uganda OR Ukraine OR Uruguay OR Uzbekistan OR Vanuatu OR Venezuela OR Vietnam OR Yemen OR Zambia OR Zimbabwe.
