# Supplementary material for: Primary palliative care in low- and middle-income countries: A systematic review and thematic synthesis of the evidence for models and outcomes
Source: Palliat Med. 2024 May 1;38(8):776–89. doi: 10.1177/02692163241248324 (PMC11487876; doi:10.1177/02692163241248324)
Supplement: sj-docx-1-pmj-10.1177_02692163241248324 – Supplemental material for Primary palliative care in low- and middle-income countries: A systematic review and thematic synthesis of the evidence for models and outcomes [file sj-docx-1-pmj-10.1177_02692163241248324.docx]

Supplemental Table 1: Study Characteristics

| **Year**  **Author**  **Setting**  **Disease Focus** | **Aims** | **Description of Care Model** | **Study Design**  **& Data collected** | **Study participants** | **Data collected** | **Relevant Findings** |
| --- | --- | --- | --- | --- | --- | --- |
| 2016 Aantjes^33^  Zambia  HIV/AIDS | To explore how community-based chronic care programmes mobilised people to help care for HIV-infected patients and, as ART became available, adjusted their services towards providing chronic care, including patient self-management support, in Zambia | Palliative and EoL care began as Christian churches and associated medical teams administering care to people dying from AIDS in the 1980s. In the 1990s, the Zambian government decentralized the healthcare system and established neighborhood health committees to coordinate primary care services. Most care was provided by volunteers in the home in conjunction with family carers. As ART became more available, the EoL care shifted to chronic care for patients with AIDS. Through the 2000s, there was a government push to revitalize primary health care services and employ medical professionals as opposed to volunteers. The ministry of health hired nurses and aids to deliver care in primary care clinics and in homes. Now, care is focused more on diagnosis and treatment adherence vs holistic care aimed at QoL improvements. | Qualitative historical comparative approach (descriptive) | 153 interviews with healthcare providers, patients, and families, focus groups, and survey respondents | Semi-structured qualitative interviews, field observations, novel survey | Long-standing presence of extensive mutual support in communities, the invocation of cultural values that emphasise social relationships and organisation of people by civil society organizations in care and support programmes. This laid the foundation for a locally conceived model of chronic care capable of addressing the new care demands arising from the country’s changing burden of disease. |
| 2020 Abboah-Offei^60^  Ghana  HIV/AIDS | To develop a community-based enhanced care intervention to improve person-centred outcomes for people living with HIV, and to test feasibility in terms of participant recruitment and retention, intervention delivery and acceptability, and estimate of potential effect to determine if a future definitive trial is warranted | Primary care services are based out of small community clinics. This intervention involved a three-day training program for primary care providers addressing holistic assessment (including physical, psychological, social, and spiritual well-being), collaborative care planning, and communication. They also received mentorship from experienced primary care providers. Patients came to the PCP 3 times over three months to receive the intervention. | Cluster randomized controlled trial | 60 healthcare providers | APOS, Medical Outcome Scale - HIV, Positive Outcomes Scale, CARE Measure, Patient Experience Questionnaire, recruitment and retention rates, fidelity, effect size, qualitative interviews | A person-centred intervention delivered in the community to improve person-centred outcomes for people living with HIV was feasible and acceptable to patients, families, and healthcare providers. Patients felt listened to, informed, respected, and involved in their care decisions, while the healthcare providers appreciated the opportunity to holistically assess symptoms and concerns. This suggests the need for a larger, definitive trial. |
| 2022 Afolabi^34^  Nigeria | To identify preferences and expectations for primary PC among people living with serious illness and their families and the readiness of primary healthcare providers to deliver primary PC in Nigeria | Nigeria has 29,360 healthcare facilities within the public health system, of which, 95.5% are primary. Nigeria is classified in Category 3a of PC development (only isolated PC provision) and has only 17 reported PC facilities (mostly tertiary hospital specialist PC teams) catering to around 190 million people. Patients and families access community based primary healthcare services most often, so they trust their PCPs and feel comfortable discussing palliative and EoL needs with them. Patients typically defer to the expertise of the healthcare provider in making decisions, and the patient centered care model is not frequently used. Staffing shortages in primary care make it difficult to provide appropriate holistic care to everyone who needs it. | Qualitative descriptive | 48 participants (21 patients, 15 family members and 12 primary healthcare providers) | Semi-structured qualitative interviews | Three major considerations for integrating PC within primary care were identified: 1. Engaging patients and families; 2. Managing patients and families’ expectations and preferences; and 3. Addressing staffing-related issues. |
| 2019 Atreya^46^  India  Cancer | To explore the facilitators and challenges in providing home‑based PC as perceived by primary care providers and family physicians | There has been a cultural shift in India over the last few decades towards accepting that palliative and EoL care is an essential part of good quality healthcare. Now many institutions are integrating PC into specialties caring for patients with chronic life‐threatening illnesses and increasing generalist and specialist outreach in the community. One of the main challenges they face in delivering care to those who need it is the lack of referral pathways from tertiary to community care. | Cross-sectional descriptive | 100 participants | A novel survey with questions related to providers' knowledge of and attitudes towards PC, training, and confidence providing care | 62% of participants reported that they were involved in palliative management of at least one cancer patient in the previous year. 34% lacked confidence in providing this care because of patient complexity, inadequate training and insufficient resources. Other barriers included poor communication from specialists and treating teams. |
| 2016 Azevedo^57^  Brazil | To characterize available services in the primary healthcare system and to explore challenges to implementing PC services within the Brazilian national healthcare network | In the Minas Gerais state of southeastern Brazil, there are 12 sectors or health regions that each contain at least one primary healthcare unit (PHCU). Each PCHU employs nurses, nurse technicians, physiotherapists, doctors, dentists, oral health technicians, and community health workers. These units typically serve an urban area and/or the surrounding countryside. They are located in adapted houses, operate from 7am to 5pm on weekdays, and are accessible to most of the population by public transport. The PHCU's offer very limited PC services - they have no protocols for PC and cannot dispense pain medication. | Cross-sectional descriptive | Data from 75,524 patients (2,715 were eligible for PC) | Diagnoses of the patients registered at the PCHU, Karnofsky Performance Scale | The current model of care does not fully meet patients' PC needs. Brazilian health policy focuses on curative treatment, so PC is often not seen as a priority. Nurses had no palliative training. |
| 2022 Beiranvand^49^  Iran  Cancer | To develop a new model of hospice care delivery in Iran, to identify priority areas within this model, and to validate the model in terms of importance, scientific acceptability, and feasibility | Currently, Iran does not have any integrated structure for the provision of hospice or end-of-life care. Some hospitals in large urban areas provide in-patient PC based solely on clinicians' experience and knowledge (there are no established clinical guidelines). The new model of care developed in this study involves developing training and defining responsibilities for healthcare professionals at different levels related to their role in delivering PC, referral protocols and pathways for primary care practitioners, and designing robust electronic medical records that can be shared across the healthcare system. | Sequential mixed methods | 18 healthcare professionals | Novel survey about the relative priority of the domains and subdomains of PC delivery | By consensus, the most important steps in establishing the hospice care delivery system were to focus on policymaking, implement public awareness programs, train and supply healthcare professionals, strengthen referral processes, formulate clinical guidelines, encourage private sector and NGO investment, and create quality care indicators |
| 2013  Budkaew^51^  Thailand  Cancer | To explore the perceptions and experiences of generalists in primary care settings about their basic knowledge of and attitudes toward PC in order to gain insight of where deficiencies in care availability and delivery can be improved | In the northeast of Thailand, primary care is delivered out of community hospitals by generalist physicians or out of primary care clinics by a multidisciplinary team. Nearly all generalist physicians receive PC training in medical school, but many felt unprepared to disclose diagnosis to patients, opioid prescription and titration, and emergency and end-stage management of terminal cancer patients. | Cross-sectional descriptive | 63 physicians | A novel survey (piloted tested/ validated) including questions about demographics and knowledge of and attitudes towards PC | Most generalists cared for less than 10 terminal patients per year. They had gaps in knowledge related to truth telling, pain control and morphine, emergency management in terminal cancer care and treatment of fluid intake in terminal stages. Positive attitude and knowledge scores were statistically correlated (p=0.036) |
| 2021 Campbell^35^  South Africa | To explore and describe PC practices of home-based carers in Limpopo Province focusing on sociocultural context affecting rural eol care, the PC practices of the home-based carers, and PC services available within the province | In many communities in South Africa, there are no specialist or generalist PC services. Community health workers, or home-based carers (lay healthcare providers who offer basic care, screening, education, psychosocial support, and referrals), help fill the gap, especially in rural communities. They are often volunteers or are paid by NGOs. | Qualitative descriptive | 34 home-based carers or professional nurses | Qualitative interviews and focus group discussions | Three major themes: the influence of the Vha-venda cultural context, home-based carers' PC practices, and the lack of PC resources & supplies. Despite a lack of resources, community health workers play an important role in PC delivery by providing physical and spiritual comfort, educating patient-family, liaising with the healthcare team, and medication management |
| 2020 Detsyk^67^  Ukraine | To evaluate quality of life in palliative patients receiving PC from family physicians, the impact of PC provided by mobile PC team on their QoL, and develop recommendations for PC provision | A mobile PC team operated out of a primary care clinic made up of family physicians, nurses, social workers, a psychologist, a driver, and medical chaplain operating out of a primary healthcare center develops a comprehensive care plan taking into account their physical, social, mental, and spiritual needs for patients and their families. They also can provide patients with medical equipment like wheelchairs, hospital beds, oxygen tanks, etc. | Cross-sectional descriptive | 219 patients | Quality of Life Questionnaire (QLQ-C30, version 3) | Patients who received general PC from a family physician had mean QoL scores of 38.63 (16.9). For patients who received PC from the mobile PC team had increased QoL scores. Mean pain, fatigue, and level of financial difficulties also decreased |
| 2005 Dumitrescu^58^  Romania  Cancer | To retrospectively evaluate the provision of PC home care from the perspective of healthcare workers after the patient’s death | PC is delivered at home delivered by multidisciplinary teams. Each team had at least a GP, an oncologist (as consultant) and one nurse. This is a relatively new model of care in Romania, where PC has traditionally been delivered in specialist hospice facilities or hospitals. | Retrospective cohort | 18 healthcare professionals working in home care (5 GPs, 4 oncologists, and 9 nurses) | Novel questionnaire with questions related to comfort with professional role, emotional burden, communication, and judgement of provided care | GP’s and nurses reported emotional burden more frequently than oncologists. Respondents felt unprepared to care for patients with unexpected complications, but that they delivered appropriate care when necessary. The multidisciplinary teams functioned well, and team communication was often satisfactory. In treating patients. Conversely, communication with patients and families was perceived as inadequate |
| 2006 Dumitrescu^55^  Romania  Cancer | To assess experience, knowledge, and opinions of Romanian GPs on PC provision in Romania | Informally, primary care physicians in Romania have been delivering PC services for years but are not reimbursed by the national healthcare system. In this representative sample of GPs, nearly all reported delivering PC services to their patients, but few felt their PC knowledge was adequate to do so effectively. When asked how PC should be delivered, most said by an interdisciplinary team in patients’ homes. | Cross-sectional descriptive | 914 GPs | Novel questionnaire with questions related to GPs’ experience with PC delivery, knowledge of PC, and opinions of the future of PC in Romania | Respondents had limited experience providing PC, with only 24% providing PC frequently.  Older age and female sex of GPs along with rural practice were associated with providing PC more frequently. Most reported that their medical knowledge was inadequate and that they did not feel comfortable providing PC services to terminal patients at home. Over 80% of GPs wanted more PC training. |
| 2007 Dumitrescu^36^  Romania  Cancer | To describe the activities and interventions carried out by an at-home PC team treating cancer patients who died within two years of being enrolled in a PC program | Nearly 90% of Romanians die at home. Home-based PC services are rare and are not able to meet the need. This paper described a model of care in which a multidisciplinary team (GPs, oncologists, nurses, and social workers) provided care in patients’ homes. | Longitudinal cohort | 102 patients | Frequency and intensity of pain, nausea, and breathlessness, visits by the PC team and interventions performed | PC care teams delivered frequent and various Interventions, and as a result, symptom frequency and intensity decreased considerably. Patients living in urban areas and with low-income experienced the most significant improvements in symptoms. |
| 2018 Gongal^52^  Nepal | To ascertain mid-level healthcare workers’ perceptions of PC and care of the dying in their local community to inform service development | Most PC services are available in the capital, Kathmandu. Most primary healthcare in rural Nepal is delivered by midlevel health workers, and they have no formal PC training. They deliver PC services in clinics and in patients’ homes out of necessity, but the lack of training and resources causes significant frustration. Some rural health workers pay for pain medications and treatments out of pocket because their patients are destitute. | Qualitative descriptive | 28 mid-level healthcare workers | Focus group discussions | Four themes emerged from the discussions: 1) suffering of patients and families resulting from life-threatening illness, 2) helplessness and frustration, 3) sociocultural issues at the end of life, and 4) improving care for patients with PC needs. Respondents felt they had limited knowledge and skills of providing PC services, having conversations around death and dying, and managing opioids. |
| 2011 Grant^37^  Uganda, Kenya, Malawi | To describe patient, family and local community perspectives on three community-based PC projects in countries with high HIV prevalence | In Malawi, the Home-based Care Charitable Trust employs nurses, care assistants, and a data manager to provide home-based PC services. This program is funded by a non-profit organization but operates out of government health centers. In Uganda, the Kitovu Mobile PC service is part of a larger faith-based community programme of HIV support which focuses on home-based care, orphans and family support. In Kenya, the Maua PC programme was based in the community health department of a rural Methodist church hospital. All three programs provide PC services for people living with HIV/AIDS and their families. | Qualitative - rapid evaluation methodology | 33 patients with advanced illness (HIV, TB, and Cancer), 27 family carers, 36 staff, 25 volunteers, and 29 community leaders | Photographs, semi-structured qualitative interviews, observation of practice, document review | In each country, oral morphine was being used effectively. Being supported at home reduced physical, emotional and financial burden. Practical support and instruction for caregivers in feeding and bathing patients facilitated good deaths at home. Staff and volunteers generally reported that caring for the dying was stressful, but also rewarding. Staff felt more resilient with effective analgesia and community support networks. |
| 2016 Hacikamiloglu^47^  Turkey  Cancer | To assess the effect of the Middle East Cancer Consortium training programs in developing and training PC practitioners in Turkey | There are few oncologists and PC specialists in many Middle Eastern countries and limited PC training for generalists. After a period of intensive training to develop a workforce of PC specialists and generalists trained in PC basics, the Turkish Ministry of Health established 407 home care teams to provide patients with essential medical treatment, including for neutropenia, high temperature, diarrhea, pressure wounds, and pain management. Trained specialists also visit government funded primary care clinics periodically to provide PC services. | Longitudinal cohort | 434 nurses, oncologists, epidemiologist, internal medicine physicians, and GPs | Novel survey evaluating participants’ knowledge of and confidence delivering PC services | This novel initiative led to a dramatic increase in the number of population-based and home-based health teams trained in PC care delivery and subsequently increased the number of cancer patients receiving PC in their homes. |
| 2021 Hojjat-Assari^48^  Iran  Cancer | To develop an integrated model of community-based PC into primary healthcare for terminally ill cancer patients | In Iran, primary healthcare is delivered out of health bases located in each small community by multidisciplinary teams with training to deliver basic PC services, and acute healthcare is delivered out of comprehensive medical centers located in large cities. When a patient is diagnosed with a life-limiting or terminal illness, they are referred by their specialist to a home base in their area with links to home-based care and socials services. When patients are close to death or experience an emergency, they are referred back to the comprehensive medical center. | Mixed methods - qualitative descriptive and Delphi survey | 21 healthcare workers and policy experts | Semi-structured qualitative interviews and a novel survey with questions related to components of community-based PC care delivery | A model of care was developed in which patients are identified as terminally ill, then are referred to the local comprehensive health center. After referral, home healthcare teams based in the primary healthcare setting deliver appropriate PC services until the patient’s death. |
| 2022 Hojjat-Assari^61^  Iran  Cancer | To explain health care providers’ perception of the integration of PC into primary healthcare | Patients with a cancer diagnosis are referred to a primary care clinic in their area who are able to deliver most palliative and end-of-life care services. If they have complex needs, they are referred to a comprehensive medical center, usually located in larger, urban areas. Most healthcare workers interviewed for this study feel this is an appropriate system that meets patients’ needs. Some felt that home-based services needed to be expanded so families can better care for dying patients at home. | Qualitative descriptive | 29 healthcare workers | Semi-structured qualitative interviews and focus group discussions | Three themes were identified: 1) the health system’s structure as an opportunity, 2) unmet needs required to integrate PC into existing primary care services, and 3) Desired outcomes including improved access to services and “good death.” |
| 2018 Jabbari^62^  Iran | To explore the feasibility of organizing palliative and end-of-life services delivered by family physicians in rural areas of Iran | The government funded health network provides healthcare services to all rural residents of Iran in teams consisting of family physicians, nurses, and behvarzes (indigenous healthcare workers with two hears of training). Those with complex needs are referred to larger, comprehensive medical centers. The Iranian health network is in the process of updating all health records to be electronic to aid in referrals. | Qualitative descriptive | 36 participants (23 GPs and 13 key stakeholders) | Semi-structured qualitative interviews | Most GPs indicated that they did not have sufficient involvement in providing PC services but emphasized the importance of PC delivery. They, along with other stakeholders agreed that GPs have the access and capacity to provide PC services in rural areas. |
| 2017 Jayalakshmi^38^  India | To understand the structure, organization, and delivery of primary PC program in rural Kerala, India and to assess its alignment with to the state's PC policy and guidelines for two local self-government Institutions | In 2008, the government of Kerala announced a community-based public health PC policy. Local self-government institutions in conjunction with primary health care clinics lead holistic care provision with assistance from NGOs in some cases. Multidisciplinary team members receive extensive training in PC, holistic care, and homeopathy. The model of care is person- and family centered and calls for shared decision making. Most care is nurse driven with supervision from physicians and support from volunteers and health aids. Some care is provided in clinics, some in patients’ homes. Some primary care clinics had visiting PC specialists offer services on specified days. All care is free to patients. | Qualitative descriptive | 378 patients | Assessment of policy and organization structures relating to PC service delivery, semi-structured qualitative interviews, field observation | Service delivery in the two sites varied considerably in terms of composition of the palliative team, Infrastructure, cost, and type of services provided. Compliance with policy guidelines was to be poor in both sites. |
| 2010 Kabore^59^  Botswana, Lesotho, Namibia, South Africa  HIV/AIDS | To document changes over time in patients’ clinical outcomes and to correlate these changes with exposure to community support programs | The team of researchers and stakeholders developed a community treatment support guidance manual and a training regimen given by teams of government, NGO, and local community-based organizations and volunteers. The model stresses the value of supportive services such as nutrition support and home-based care to help those living with HIV/AIDS manage their disease. Patients are served in primary care clinics and at home. All services were provided by experienced and trained healthcare workers, community workers and volunteers. | Prospective, observational cohort | 377 patients | Health-related quality of life (including physical, cognitive, emotional, and social functioning), treatment adherence, CD4 cell counts | Those exposed to community-based supportive services experienced a more rapid and greater overall increase in CD4 cell counts and better treatment adherence than unexposed patients. Those receiving home-based care and/or food support services showed greater improvements in selected health-related quality of life indicators. |
| 2018 Kitreerawutiwong^50^  Thailand | To develop a community-based PC model in a district health system | Thailand has limited PC development. Home-based PC services are rare, and most services are provided at tertiary hospitals and cancer centers by public facilities, private hospitals, and faith-based institutions. In the model described in this article, primary care is offered out of community hospitals. Generalists at the community hospital developed PC guidelines, a PC training program for healthcare workers, and provided a care manager to help coordinate home care delivered by “formal caregivers” paid by the hospital for patients with life limiting illness. | Mixed methods action research | 41 patients | Semi-structured qualitative interviews, Activities of daily living, accessibility and continuity of PC services, and caregiver burden | Respondents reported confusion with PC terminology, the challenge of the referral system, a lack of medical equipment and supplies for PC patients, and insufficient access to opioid analgesics at home. The developed model involved PC training for healthcare workers, better management of medical equipment and supplied, and referral guidelines. This new model improved accessibility and continuity of PC services. |
| 2022 Kochuvilayil^65^  India | To assess the proportion of female caregivers who screen positive for depression and to explore the associated factors | The PC nurse is a part of the primary health care team in all parts of Kerala and makes regular visits to registered bedridden and home-bound patients. The nurse is usually supported by Accredited Social Health Activist (ASHA) workers, other field workers and often trained volunteers | Cross-sectional cohort | 40 caregivers | Primary Care Screening Questionnaire for Depression - Quality of Life Domain | Almost half of the female caregivers had at least one medical condition needing regular treatment. About 20% screened positive for depression Spousal relation, a diagnosis of cancer in the patient, and moderate to high caregiver burden, and high financial burden were associated with a higher likelihood of depression. |
| 2019 Lai^39^  China  Cancer | To explore how home-based end-of-life care is delivered in community health service centers in Shanghai and to examine challenges PC care delivery in this setting | A small proportion of people receive home-based end-of-life care if eligible (advanced stage cancer or less than one year life expectancy, stable enough to be managed at home, but with distressing symptoms). They receive home -based (home visits and telephone support) from physicians and nurses based out of at community health centers. Sometimes, social workers, psychologists, and volunteers are included in the care team. Few members of the team have any PC training. Patients were sometimes classed as “hospitalized at home” and were given the same treatment as hospitalized patients. | Qualitative descriptive | 16 healthcare providers | Semi-structured interviews | Four major themes were identified in analysis: 1) Patient description – most patients has less than one year life expectancy, 2) Service structure – mostly delivered by nurses and physicians through home visits and phone calls, 3) Service process – involved monitoring and treating symptoms, nursing care, and psychological support, and 4) Difficulties in delivering care – resource limitations and feeling powerless facing psycho-spiritual issues. |
| 2017 Pereira^40^  Brazil | To understand the perceptions of nurses and managers in primary health care settings related to palliative and end-of-life care | There is no national PC policy in Brazil. Nurses based at primary health care units deliver holistic PC to patients and families both in clinics and in homes. They operate within a multidisciplinary primary care teams and make referrals to other specialties with necessary. The receive extensive PC training. | Qualitative descriptive | 15 nurses and 3 managers | Semi-structured interviews. | Four main themes were identified: 1) Intersection of PC, terminality, and cancer, 2) the importance of QoL preservation, 3) Different meanings attributed to PC, and 4) Association between professional knowledge of PC and quality of care. Participants had insufficient knowledge of PC but understood the importance of maintaining quality of life. PC training is lacking. |
| 2019 Potts^66^  India  Cancer | To evaluate feasibility, utility and acceptability of a home-based PC program from the perspectives of the clinical team and CHWs | In India, there are not enough trained healthcare professionals (physicians, nurses, etc) to meet the need. To reach more people, this study reports a model that provides home-based PC services for under-resourced rural areas delivered by unlicensed rural medical practitioners and community healthcare workers (with varying levels of training). They provide pain and symptom management, basic nursing tasks, and referrals to other available services when needed. | Qualitative descriptive | 10 participants (7 clinical team members 3 CHWs) | Semi-structured interviews | Three major themes emerged: 1) CHWs’ desire and need for more training, 2) Need for clear protocols and expectations for stakeholders, 3) Sustainability of the home-based PC program. Given enough training and support, CHW's would be an acceptable workforce for delivering PC to stretch resources. |
| 2021 Rodrigues Quintana^41^  Ecuador | To assess implementation of the Ecuadoran Ministry of Public Health’s PC guide in selected health centers and to assess healthcare professionals’ level of knowledge about PC six years after approval of the PC National Plan. | Only 3.5% of PC need is met in Ecuador. In 2014, the Ministry of Public Health approved the Clinical Practice Guide for PC and made it mandatory in the national health system. Health centres in urban and rural areas staffed by physicians and nurses provide most primary healthcare services. PC training is mandatory for these professions, but gaps in knowledge and skills exist. | Cross-sectional descriptive | 292 healthcare professionals (38 managers, 150 physicians, 104 nurses) | Novel surveys to assess PC knowledge and implementation of the national PC guide, medical record review | More than half of the participants had no training in PC. Implementation of the PC guide was inadequate in 52.9% of patients’ cases, highlighting the lack of information about the guide, training on its application and limited availability of opioids. Health professionals felt they lacked specific PC training, particularly in the use of morphine and its secondary effects and in the sphere of spirituality. |
| 2019 Shi^53^  China | To investigate community healthcare providers’ the knowledge of and attitudes toward EoL care and to provide reference for developing effective strategies to promote end-of-life care in China. | EoL care was established in China in the 1980s, but development has been slow. Nurses and community healthcare workers provide the majority of EoL care out of community health centers. They receive little to no PC training. Most interviewed in this study had positive attitudes toward EoL care, but they lacked knowledge and skills to care for terminal patients. | Cross-sectional descriptive | 132 community doctors and nurses | Novel survey assessing knowledge of and attitude toward EoL care, experience caring for the dying, Bradley Attitude Questionnaire and Death Attitude Profile - Revised | Participants had positive attitudes toward EoL care, but they lacked professional knowledge and skills to appropriately care for terminal patients, particularly in communication and pain management. Those who had worked longer, had personal experience with the death of a loved one, or had previous experience caring for terminal patients had more positive attitudes toward caring for the dying. |
| 2021 Silva^64^  Brazil | To assess PC services offered to home care patients I the national care system of Brazil | Brazil has no national PC policy. They do have guidelines for how home-based healthcare services should meet the needs of patients who are dying, including effective symptom control, targeted communication, family support, and preparation for home death. Multidisciplinary teams of nurses, doctors, speech therapists, physiotherapist and nursing technicians deliver home-based care. | Qualitative descriptive | 13 healthcare professionals (4 nurses, 3 doctors, 1 speech therapist, 1 physiotherapist, 4 nursing technicians) | Semi-structured qualitative interviews, field observations | Participants provided limited PC services, like basic symptom management, open communication, carrying out national care guidelines, education, and referral to specialists. The actions of the participating healthcare professionals were found to fall short of the humanistic principles of PC as recommended by the WHO which highlights the need for advances in PC policy in Brazil. |
| 2021 Siva^42^  India  Cancer | To explore experiences of community health nurses in PC delivery in a primary health care setting | In this study, community health nurses delivered primary PC services in patients’ homes. They receive basic training in PC. The service is mainly funded by the government and non-governmental organizations. | Qualitative descriptive | Not stated | Qualitative interviews and focus group discussions | Analysis of transcripts identified five major themes: 1) community support, 2) family support, 3) acceptance of services, 4) barriers and 5) gaps in care. Focus groups showed that community health nurses can deliver holistic care in primary care setting in collaboration with secondary and tertiary care centers to improve outcomes. |
| 2004 Tache^54^  Bosnia-Herzegovina  Cancer | To identify major obstacles encountered by GP's and nurses providing end-of-life care | PC services are severely lacking in Bosnia-Herzegovina. General practice doctors and nurses provide end-of-life care, and most patients die at home. Most health professionals receive no PC training. Very few interviewed in this study felt confident controlling patients’ pain or informing the patient of their terminal diagnosis. | Cross-sectional descriptive | 130 physicians and nurses | Novel survey (pilot tested) with questions related to frequency of home visits, level of PC training, ability to relieve pain, and communication | Most participants performed home visits on a weekly basis. And almost half had visited 2-3 terminally ill cancer patients in the previous month. Very few had any PC training. Most ranked pain control as first priority in providing PC, but few felt they did so effectively. Most only discussed terminal diagnosis with a patient’s family, not the patient. |
| 2012 Thayyil^43^  India | To assess patient's status, in relation to indicators of QoL and activities of daily living, the services provided by palliative home care, and the degree of unmet need | Local self-governments dictate PC policy in Kerala, India. Community home-based PC services are delivered by a team of nurses, health volunteers, accredited social health activist and community members, supported by field workers from the Department of Health and emergency doctors from a medical college training centre. | Retrospective cohort study | 104 patients | Physical, psychological, and social aspects of care which indicate QoL, patient/ family needs, and provided services | The home-based PC programme was a cost-effective method of addressing most of the medical, psychosocial and supportive needs of patients as well as reducing pain and other symptoms. |
| 2001 Uys^63^  South Africa  HIV/AIDS | To describe the implementation of the integrated community-based home care model in seven sites in South Africa in terms of implementation, problems, client profile and utilisation | Integrated community-based HIV/AIDS home care is delivered out of community health clinics. They aim to provide a continuum of care in patients’ homes including counselling and support of people with AIDS and their caregivers from diagnosis to death and bereavement. Components of the program include trained Community Caregiver Teams, paid volunteers from disadvantaged areas, and a senior nurse as hospital project coordinator at each participating provincial hospital. | Mixed methods descriptive | Not specified | Qualitative interviews, focus group discussions, field observations, service utilization gathered from clinics | This integrated home health model conforms to the guidelines set forth by the Dept. Of Health. It is an economical model for delivering quality care. |
| 2022 Vanheerden^44^  South Africa | To explore the roles of CHWs in PC delivery in a rural subdistrict in South Africa | South Africa’s National Policy Framework and Strategy on PC identifies that most people receive PC services in their community in primary care. Nurses, community health workers, and home-based carers provide the majority of PC services in patients’ homes, in primary care clinics, and in a district hospital. They are funded by non-governmental organizations and the local department of health. | Qualitative descriptive | 39 total (23 CHW's, 5 CHW supervisors, 1 policy implementer, 5 patients, 5 primary caregivers) | Semi-structured qualitative interviews and focus group discussions | Four themes were identified: 1) The need for PC in the community, 2) CHW's are unsure of their roles and responsibilities in PC, 3) Barriers to CHW's roles in PC and pain management, 4) Enablers to CHW's roles in PC and pain management. Findings supported the need for community-based PC and highlighted aspects of the roles of CHW's and their ability to contribute to effective PC. |
| 2019 Yennurajalingam^56^  Ghana, Zambia, Kenya, Nigeria,  Tanzania, South Africa  Cancer | To describe the development of the Project Extension for Community Healthcare Outcomes PC in Africa (ECHO-PACA) program and describe attitudes and knowledge of participants regarding the ability of the program to deliver quality PC | Rural primary care providers receive PC training, mentoring, and support from PC specialists over Zoom. They form a network of providers and can ask for help with their most difficult cases. These providers can then provide basic PC services to patients in their rural community. | Longitudinal cohort study | 40 participants | Surveys with questions related to knowledge of and attitudes towards assessing and managing pain, and communication | Practitioners who participated in the ECHO-PACA programme reported an increase in confidence that they could deliver quality PC, with particular respect to titrating opioids for pain control, use of non-opioid analgesics and addressing communication issues related to end-of-life care. |
| 2022 Zhang^45^  China | To explore the challenges and obstacles faced by community nurses delivering home based hospice and PC | Home based hospice and PC is delivered by community nurses, general practitioners, pharmacists, and rehabilitation therapists either in hospitals (not covered by health insurance) or in patients’ homes (free). Healthcare workers often receive limited PC training. | Phenomenological Qualitative descriptive | 13 nurses | Semi-structured qualitative interviews | Three major themes highlighting challenges to nurses delivering home-based PC were identified: 1) Inadequate preparation for providing care, 2) patients and families' non-cooperation, 3) career disadvantages. Authors concluded that better self-preparation and increased organisational support for community nurses undertaking home-based PC is necessary. |

Abbreviations – APOS: African Palliative Outcome Scale, CHWs: community health workers, EOL: end of life, GP: general practitioner, PC: palliative care, QoL: quality of life, WHO: World Health Organization

Supplemental Table 2. Quality of the Evidence

| **Studies** | **Criteria from Mixed Methods Appraisal Tool** | | | | | | | | | | | | | | | | | | | | | | | | | |
| --- | --- | --- | --- | --- | --- | --- | --- | --- | --- | --- | --- | --- | --- | --- | --- | --- | --- | --- | --- | --- | --- | --- | --- | --- | --- | --- |
|  | **1.1** | **1.2** | **1.3** | **1.4** | **1.5** | **2.1** | **2.2** | **2.3** | **2.4** | **2.5** | **3.1** | **3.2** | **3.3** | **3.4** | **3.5** | **4.1** | **4.2** | **4.3** | **4.4** | **4.5** | **5.1** | **5.2** | **5.3** | **5.4** | **5.5** | **Total** |
| 2016 Aantjes^33^ | 1 | 1 | 1 | 1 | 1 |  |  |  |  |  |  |  |  |  |  |  |  |  |  |  |  |  |  |  |  | 5 |
| 2020 Abboah-Offei^60^ |  |  |  |  |  | 1 | 1 | 1 | 0 | 1 |  |  |  |  |  |  |  |  |  |  |  |  |  |  |  | 4 |
| 2022 Afolabi^34^ | 1 | 1 | 1 | 1 | 1 |  |  |  |  |  |  |  |  |  |  |  |  |  |  |  |  |  |  |  |  | 5 |
| 2019 Atreya^46^ |  |  |  |  |  |  |  |  |  |  |  |  |  |  |  | 0 | 0 | 1 | 0 | 1 |  |  |  |  |  | 2 |
| 2016 Azevedo^57^ |  |  |  |  |  |  |  |  |  |  |  |  |  |  |  | 1 | 1 | 0 | 1 | 0 |  |  |  |  |  | 3 |
| 2022 Beiranvand^49^ | 1 | 1 | 1 | 0 | 0 |  |  |  |  |  |  |  |  |  |  | 1 | 0 | 1 | 0 | 1 | 1 | 1 | 1 | 0 | 1 | 3 |
| 2013 Budkaew^51^ |  |  |  |  |  |  |  |  |  |  |  |  |  |  |  | 1 | 0 | 1 | 0 | 1 |  |  |  |  |  | 3 |
| 2021 Campbell^35^ | 1 | 1 | 1 | 1 | 1 |  |  |  |  |  |  |  |  |  |  |  |  |  |  |  |  |  |  |  |  | 5 |
| 2020 Detsyk^67^ |  |  |  |  |  |  |  |  |  |  |  |  |  |  |  | 1 | 1 | 0 | 1 | 1 |  |  |  |  |  | 4 |
| 2005 Dumitrescu^58^ |  |  |  |  |  |  |  |  |  |  |  |  |  |  |  | 1 | 1 | 0 | 0 | 1 |  |  |  |  |  | 3 |
| 2006 Dumitrescu^55^ |  |  |  |  |  |  |  |  |  |  |  |  |  |  |  | 1 | 0 | 1 | 1 | 1 |  |  |  |  |  | 4 |
| 2007 Dumitrescu^36^ |  |  |  |  |  |  |  |  |  |  |  |  |  |  |  | 1 | 1 | 1 | 1 | 1 |  |  |  |  |  | 5 |
| 2018 Gongal^52^ | 1 | 0 | 1 | 1 | 0 |  |  |  |  |  |  |  |  |  |  |  |  |  |  |  |  |  |  |  |  | 3 |
| 2011 Grant^37^ | 1 | 1 | 1 | 1 | 1 |  |  |  |  |  |  |  |  |  |  |  |  |  |  |  |  |  |  |  |  | 5 |
| 2016 Hacikamiloglu^47^ |  |  |  |  |  |  |  |  |  |  |  |  |  |  |  | 1 | 1 | 0 | 0 | 0 |  |  |  |  |  | 2 |
| 2021 Hojjat-Assari^48^ | 1 | 1 | 1 | 1 | 1 |  |  |  |  |  |  |  |  |  |  | 0 | 1 | 1 | 1 | 1 | 1 | 1 | 1 | 0 | 1 | 4 |
| 2022 Hojjat-Assari^61^ | 1 | 1 | 1 | 1 | 0 |  |  |  |  |  |  |  |  |  |  |  |  |  |  |  |  |  |  |  |  | 4 |
| 2018 Jabbari^62^ | 1 | 0 | 1 | 1 | 0 |  |  |  |  |  |  |  |  |  |  |  |  |  |  |  |  |  |  |  |  | 3 |
| 2017 Jayalakshmi^38^ | 1 | 1 | 1 | 1 | 1 |  |  |  |  |  |  |  |  |  |  |  |  |  |  |  |  |  |  |  |  | 5 |
| 2010 Kabore^59^ |  |  |  |  |  |  |  |  |  |  |  |  |  |  |  | 1 | 1 | 1 | 0 | 1 |  |  |  |  |  | 4 |
| 2018 Kitreerawutiwong^50^ | 1 | 1 | 1 | 0 | 1 |  |  |  |  |  |  |  |  |  |  | 0 | 0 | 1 | 1 | 1 | 1 | 1 | 1 | 0 | 1 | 3 |
| 2022 Kochuvilayil^65^ |  |  |  |  |  |  |  |  |  |  |  |  |  |  |  | 1 | 1 | 1 | 0 | 1 |  |  |  |  |  | 4 |
| 2019 Lai^39^ | 1 | 1 | 1 | 1 | 1 |  |  |  |  |  |  |  |  |  |  |  |  |  |  |  |  |  |  |  |  | 5 |
| 2017 Pereira^40^ | 1 | 1 | 1 | 1 | 1 |  |  |  |  |  |  |  |  |  |  |  |  |  |  |  |  |  |  |  |  | 5 |
| 2019 Potts^66^ | 1 | 0 | 1 | 0 | 1 |  |  |  |  |  |  |  |  |  |  |  |  |  |  |  |  |  |  |  |  | 3 |
| 2021 Rodrigues Quintana^41^ |  |  |  |  |  |  |  |  |  |  |  |  |  |  |  | 1 | 1 | 1 | 1 | 1 |  |  |  |  |  | 5 |
| 2019 Shi^53^ |  |  |  |  |  |  |  |  |  |  |  |  |  |  |  | 1 | 1 | 1 | 0 | 1 |  |  |  |  |  | 4 |
| 2021 Silva^64^ | 1 | 1 | 1 | 0 | 0 |  |  |  |  |  |  |  |  |  |  |  |  |  |  |  |  |  |  |  |  | 3 |
| 2021 Siva^42^ | 1 | 1 | 1 | 1 | 1 |  |  |  |  |  |  |  |  |  |  |  |  |  |  |  |  |  |  |  |  | 5 |
| 2004 Tache^54^ |  |  |  |  |  |  |  |  |  |  |  |  |  |  |  | 1 | 0 | 0 | 1 | 1 |  |  |  |  |  | 3 |
| 2012 Thayyil^43^ |  |  |  |  |  |  |  |  |  |  |  |  |  |  |  | 1 | 1 | 1 | 1 | 1 |  |  |  |  |  | 5 |
| 2001 Uys^63^ | 1 | 0 | 1 | 1 | 0 |  |  |  |  |  |  |  |  |  |  | 1 | 1 | 0 | 1 | 1 | 0 | 0 | 1 | 1 | 1 | 3 |
| 2022 Vanheerden^44^ | 1 | 1 | 1 | 1 | 1 |  |  |  |  |  |  |  |  |  |  |  |  |  |  |  |  |  |  |  |  | 5 |
| 2019 Yennurajalingam^56^ |  |  |  |  |  |  |  |  |  |  |  |  |  |  |  | 1 | 1 | 0 | 1 | 1 |  |  |  |  |  | 4 |
| 2022 Zhang^45^ | 1 | 1 | 1 | 1 | 1 |  |  |  |  |  |  |  |  |  |  |  |  |  |  |  |  |  |  |  |  | 5 |
